# High-Quality Draft Genome Sequence of *Xanthomonas alfalfae* subsp. *alfalfae* Strain CFBP 3836

**DOI:** 10.1128/genomeA.01035-13

**Published:** 2013-12-12

**Authors:** M. A. Jacques, S. Bolot, E. Charbit, A. Darrasse, M. Briand, M. Arlat, L. Gagnevin, R. Koebnik, L. D. Noël, P. Portier, S. Carrère, T. Boureau

**Affiliations:** INRA, Institut de Recherche en Horticulture et Semences (IRHS), UMR 1345 SFR 4207 QUASAV, Beaucouzé, Francea; Université d’Angers, Institut de Recherche en Horticulture et Semences (IRHS), UMR 1345 SFR 4207 QUASAV, Beaucouzé, Franceb; Agrocampus Ouest, Institut de Recherche en Horticulture et Semences (IRHS), UMR 1345 SFR 4207 QUASAV, Beaucouzé, Francec; INRA, Laboratoire des Interactions Plantes Micro-Organismes (LIPM), UMR 441, Castanet-Tolosan, Franced; CNRS, Laboratoire des Interactions Plantes Micro-Organismes (LIPM), UMR 2594, Castanet-Tolosan, Francee; Université de Toulouse, Université Paul Sabatier, Toulouse, Francef; UMR “Peuplements Végétaux et Bioagresseurs en Milieu Tropical” (PVBMT), CIRAD, Saint-Pierre, La Réunion, Franceg; UMR 186 IRD-Cirad-Université Montpellier 2 “Résistance des Plantes aux Bioaggresseurs,” Montpellier, Franceh

## Abstract

We report the high-quality draft genome sequence of *Xanthomonas alfalfae* subsp. *alfalfae* strain CFBP 3836, the causal agent of bacterial leaf and stem spot in lucerne (*Medicago sativa*). Comparative genomics will help to decipher the mechanisms provoking disease and triggering the defense responses of this pathogen of the model legume *Medicago truncatula*.

## GENOME ANNOUNCEMENT

*Xanthomonas alfalfae* subsp. *alfalfae* (formerly *Xanthomonas axonopodis* pv. alfalfae) is the causal agent of bacterial leaf and stem spot of lucerne (*Medicago sativa*) (1, 2). While lucerne is the main host of *X. alfalfae* subsp. *alfalfae*, other hosts, such as soybean (*Glycine max*), clover (*Trifolium* spp.), and vetch (*Vicia* spp.), have been reported (3). *X. alfalfae* subsp. *alfalfae* is also pathogenic on the model legume *Medicago truncatula* (4). Lesions first appear on the leaf as water-soaked spots surrounded by a diffuse chlorotic area, which then turns dry, yellow-brown, and papery. Severe defoliation is a common result of leaf infection. Postemergence damping off, stunting of seedlings, and damage of lucerne stands are other symptoms. This seed-borne disease may cause severe losses in hot, moist environments (3). To better understand the molecular basis of legume-bacterium interactions, we sequenced the genome of the *X. alfalfae* subsp. *alfalfae* strain CFBP 3836. This strain is the pathotype strain of the former pathovar *X. axonopodis* pv. alfalfae (5). This strain originates from the Sudan (http://www.angers-nantes.inra.fr/cfbp/resultnum_e.php?r0=3836).

This genome was sequenced using the Illumina HiSeq 2000 platform (GATC Biotech, Germany). Shotgun sequencing yielded 116,576,062 read pairs (37,799,928 100-bp paired-end reads with an insert size of ca. 250 bp and 78,776,134 bp mate-pair reads with an insert size of approximately 3 kb). A combination of Velvet (6), SOAP*denovo*, and SOAP Gapcloser (7) yielded 22 contigs >500 bp (N_50_, 763,181 bp), with the largest contig being 2,433,808 bp, for a total assembly size of 5,077,532 bp. Multilocus sequence analyses of seven housekeeping genes described earlier for xanthomonads confirmed that strain CFBP 3836 belongs to *X. alfalfae* subsp. *alfalfae* (8).

The genome carries the core characteristics shared by most plant-pathogenic xanthomonads, including chemotaxis- and flagellum-encoding genes, many genes encoding two-component systems, and TonB-dependent transporters. This genome sequence displays the genes coding for the six secretion systems (from T1SS to T6SS) that have been identified so far in Gram-negative bacteria and multiple effectors. A minimum repertoire of 23 type III effectors is predicted. It comprises two putative transcription activator-like effectors, the highly repetitive sequence of which could not be completely resolved with this sequencing strategy. This genome sequence will be a valuable tool for transcriptomic and genetic studies to allow for a better understanding of disease development and plant defense triggering in legume-bacterium interactions.

### Nucleotide sequence accession numbers.

This whole-genome shotgun project has been deposited in GenBank under the accession no. AUWN00000000. The version described in this paper is the first version, AUWN01000000.
